# Effects of Ce, Sm and Yb on cavitation erosion of NAB alloy in 3.5% NaCl solution

**DOI:** 10.1016/j.ultsonch.2022.106093

**Published:** 2022-07-12

**Authors:** D.G. Li, S.L. Song, D.R. Chen, P. Liang

**Affiliations:** State Key Laboratory of Tribology, Tsinghua University, Beijing 100084, China

**Keywords:** NAB alloy, Cavitation erosion, Rare earth element, Metallographic structure

## Abstract

The influences of Ce, Sm and Yb on cavitation erosion of NAB alloy in 3.5% NaCl solution are evaluated using mass loss, SEM, 3D morphology and Tafel plot, respectively. The results show that the addition of Ce or Sm or Yb enhances the mechanical property of NAB alloy, and the sizes of κ_Ⅰ_ and κ_Ⅱ_ phases within NAB alloy decrease with adding Ce or Sm or Yb, resulting in the prevention of the propagation of the cracks caused by cavitation erosion initially originated at the phase boundaries between α and κ phases, and finally the cavitation erosion damage significantly decreases with adding Ce or Sm or Yb. The corrosion of NAB alloy in 3.5% NaCl solution can promote the cavitation erosion of NAB alloy, while the corrosion resistance of NAB alloy increases with the addition of Ce or Sm or Yb, and then the cavitation erosion resistance is accordingly improved with the addition of rare earth element.

## Highlights

• The grain size of NAB alloy can become fine with adding Ce or Sm or Yb.
• The decreased size of κ phase with adding Ce or Sm or Yb can reduce the stress between κ phase and the substrate α or β phase.
• NAB alloy containing Yb behaves the strongest cavitation erosion resistance in 3.5% NaCl solution.

## 1 Introduction

Cavitation erosion is a very common and serious hydraulic phenomenon which widely occurs in the hydrodynamic components including pump, valve, marine propeller, pipe elbow, tee and reducer, et al. In high-velocity water, micro-bubbles can be formed in low-pressure regions. When micro-bubbles move to high-pressure regions, they will grow and collapse, and then leading to a repeat strike of shock wave or microjet on material surface nearby, and this is called cavitation erosion [1]. As shock wave or microjet induced by the collapsed bubbles has a high velocity of several hundreds to thousands of meters per second, and the material surface will suffer a serious damage in such extreme condition [2], [3], [4]. Except the damage caused by cavitation erosion, the noise originated from the cavitation erosion during propeller rotating also heavily threatens the concealed property of submarine because the continued frequency band noise caused by cavitation erosion can easily expose the position of submarine. Therefore, it is vital to decrease the cavitation erosion noise of propeller for underwater weapon, and tremendous studies have focused on the improvement of the cavitation erosion resistance of alloys [5], [6], [7], [8], [9], [10], [11]. However, up to now, an agreed mechanism and an effective method for improving cavitation erosion resistance are not nevertheless reached.

**Fig. 1:**
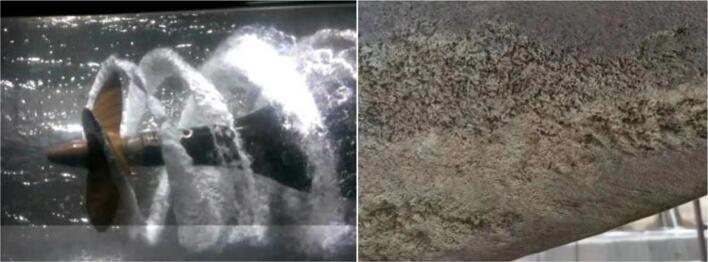
Damage caused by cavitation erosion of ship propeller made of NAB alloy in service.

Usually, nickel aluminum bronze (NAB) alloy is extensively used to manufacture the ship propeller due to its excellent erosion-corrosion resistance in seawater and good mechanical properties [12], [13], [14], [15]. However, the propeller made of NAB alloy will still suffer serious cavitation erosion damage in the actual environment, as shown in Fig. 1, the serious cavitation erosion damage occurs at the tip of propeller made of NAB alloy, which indicates that the cavitation corrosion resistance of NAB alloy in seawater is still unsatisfied. Generally, the ship propellers are manufactured by as-cast NAB alloy, and then the cavitation erosion resistance of NAB alloy in seawater is closely related to its metallographic structure. However, the as-cast NAB alloy is comprised of α phase, residual β phase and four forms of κ phases [16], [17]. Thus, the phase boundaries and the corrosion potential difference between the different phases are the origin of the corrosion and cavitation erosion [18], [19]. While, the present solutions of corrosion and cavitation erosion mainly focus on the surface modification methods including friction stir processing (FSP) [20], [21], [22], laser surface melting [23], [24] and high velocity oxy-fuel spray [25] (HVOF), et al. Although coatings and other surface modification processes are verified to be effective to improve the resistance of cavitation erosion, however, the surface treatment methods are costly and inefficient for large-scale ship propeller. Also, with the extension of time, the surface protection will gradually degrade. Therefore, the basic way to improve the cavitation erosion resistance of NAB alloy lies in NAB alloy itself.

Rare earth element is a kind of monosodium glutamate additive in metallurgical industry, which can effectively refine grains and reduce the content of impurities such as oxygen and phosphorus in the alloys, and then significantly enhancing the comprehensive performance of the substrate [26], [27], [28]. However, as we can know, there is no paper focusing on the rare earth element effect on the cavitation erosion and corrosion of NAB alloy in seawater. Herewith, four kinds of NAB alloys (without containing rare earth element, containing Ce or Sm or Yb) are manufactured in this work, then, the cavitation erosion and corrosion behaviors of four NAB alloys are investigated in 3.5% NaCl solution.

## 2 Experimental section

### 2.1 Sample preparation

The samples are prepared by melting the mixture of pure Cu (99.9% wt%), pure Al (99.9 wt%), pure Ni (99.9% wt%), pure Fe (99.9% wt%), pure Mn (99.9% wt%) and pure rare earth elements of Ce, Sm and Yb in a 100-kilogram vacuum arc remelting (VAR) furnace. The chemical compositions of the as-cast NAB samples are analyzed using chemical analysis, and the results are showed in Table 1. Before each experiment, the test surface of NAB sample is polished using 600#, 2000#, and 7000# grit silicon carbide (SiC) abrasive papers, and then the test surface is cleaned with double-distilled water and methanol before blow-drying them. All electrochemical experiments are carried out three times in parallel, and the averaged parameters are used.

**Table 1:** Chemical Composition of the manufactured NAB alloys (wt.%).

Order	Al	Mn	Fe	Ni	Re	Cu
Sample 1	9.25	1.35	4.6	4.4	0	Bal.
Sample 2	9.24	1.32	4.58	4.39	0.03Ce	Bal.
Sample 3	9.26	1.33	4.55	4.42	0.028Sm	Bal.
Sample 4	9.27	1.34	4.59	4.38	0.031Yb	Bal.

### 2.2 Cavitation erosion equipment

**Fig. 2:**
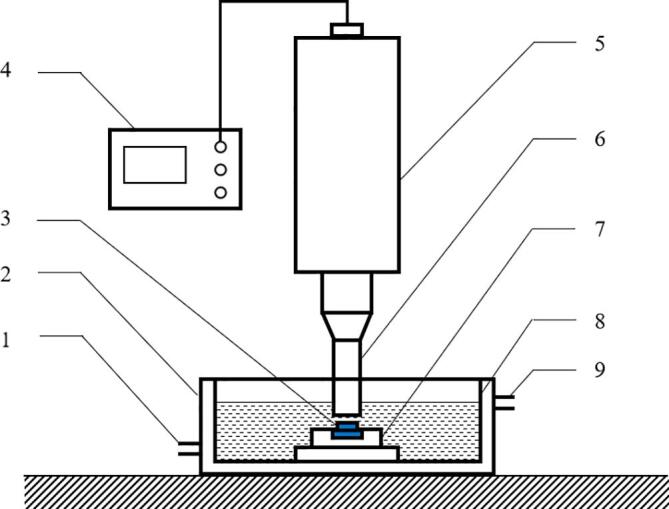
Schematic of cavitation erosion apparatus, 1: water inlet; 2: cooling bath; 3: specimen; 4: ultrasonic generator; 5: transducer; 6: horn; 7: specimen holder; 8: beaker; 9: water outlet.

Cavitation erosion is carried out according to ASTM G32 standard by an ultrasonic cavitation erosion apparatus (NingBo scientz Biotechnology Co., ltd.), which resonates at 20 KHz with the amplitude of 50 μm. The power of this apparatus is 3KW, and the schematic of the ultrasonic cavitation erosion equipment is showed in Fig. 2. In which, the sample is held in a fixture and the test surface is kept at a distance of 0.5 mm down from the bottom of the horn tip. The horn tip is submerged about 15 mm in 3.5 wt% NaCl solution kept at 5 ℃ by a cooling bath. The cavitation erosion apparatus will stop running for 1 h after the cavitation erosion test for 1 h for cooling treatment. Similar to electrochemical experiment, cavitation erosion experiments are also carried out three times in parallel, and the averaged mass loss is used.

### 2.3 Mass loss, electrochemical and surface observation experiments

The mass loss is obtained by weighing the sample mass before and after cavitation erosion test using an electronic balance with an accuracy of 0.001 mg, and each weighing is carried out three times in parallel, and finally the average of three weighed mass is used.

The microstructure of NAB alloy is observed by optical microscopy and a field emission scanning electron microscopy (SEM), and the surface morphology after the cavitation erosion test is observed by SEM and a 3-dimension surface profiler.

The electrochemical experiments are carried on the EG＆G M273A electrochemical work station with a conventional three-electrode cell with a saturated calomel electrode (SCE) as a reference electrode and the platinum sheet as an counter electrode. The as-cast NAB sample mounted in resin with an exposed area of 0.7856 cm^2^ is the working electrode. The test solution is 3.5 wt% NaCl. All potentials are measured against a saturated calomel electrode and at ambient temperature. Prior to the electrochemical tests, the samples are immerged in 3.5 wt% NaCl solution for 0 day, 14 days and 28 days, respectively.

## 3 Results and discussions

### 3.1 Influences of Ce, Sm and Yb on the metallographic structure of NAB alloy

**Fig. 3:**
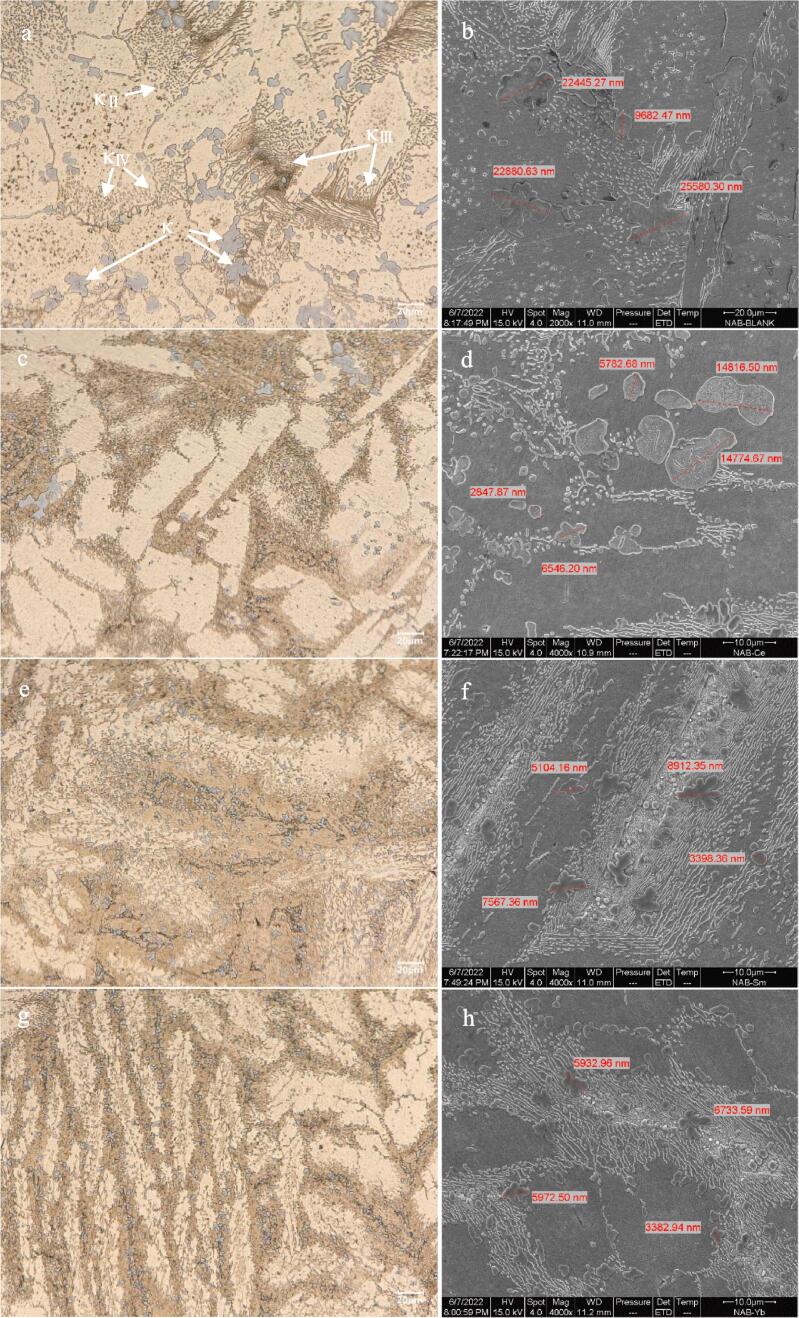
Metallographic structures of NAB alloy (a and b), NAB alloy containing Ce (c and d), NAB alloy containing Sm (e and f) and NAB alloy containing Yb (g and h).

The metallographic structures of NAB alloy without containing rare earth element and NAB alloy containing Ce or Sm or Yb are detected using optical microscopy and scanning electron microscope (SEM), respectively. The microstructures of four NAB samples are showed in Fig. 3, in which the images showed in Fig. 3a, 3c, 3e and 3 g are obtained using optical microscopy, and the images showed in Fig. 3b, 3d, 3f and 3 h are obtained by SEM. It can be seen from Fig. 3a that the metallographic structure of NAB alloy without adding rare earth element is mainly composed of the α phase (the columnar and bright part), residual β phase (the dark part) and four forms of κ phases, and four κ phases mainly disperse randomly in the α phase and β phases [16], [17]. As shown in Table 2, κ phases are essentially similar in chemical composition including the rosette κ_Ⅰ_, the globular κ_Ⅱ_, the lamellar κ_III_ and fine globular κ_Ⅳ_ phases [18]. Fig. 3b shows the corresponded microstructure of NAB alloy obtained by SEM, and it shows that the size of κ_Ⅰ_ is about 22 μm, and the size of κ_Ⅱ_ is about 10 μm. Similarly, the sizes of κ_Ⅰ_ and κ_Ⅱ_ phases showed in Fig. 3d are about 14 μm and 5 μm, and the sizes of κ_Ⅰ_ and κ_Ⅱ_ phases showed in Fig. 3f are about 7 μm and 3 μm, and the sizes of κ_Ⅰ_ and κ_Ⅱ_ phases showed in Fig. 3h are about 6 μm and 2 μm, respectively. Apparently, the sizes of κ_Ⅰ_ and κ_Ⅱ_ phases reduce with adding Ce or Sm or Yb. Considering the differences of corrosion potential at the grain boundaries between κ phase and α phase, and the cracks caused by cavitation erosion initially originates from the boundaries between κ phase and α phase, therefore, the number, morphology and distribution of κ phase play an important role in mechanical property, corrosion behavior and cavitation erosion of NAB alloy [14], [15]. The previous papers report that the cavitation erosion stress, cracks and corrosion are easy to initiate from the phase boundaries between α and κ phases [29], [30], [31], the decreased size of κ phase means the decreased cavitation erosion stress, crack and corrosion driving force, and finally resulting in the improvement of the cavitation erosion and corrosion resistances of NAB alloy.

**Table 2:** Chemical composition of constituent phase of NAB alloy (ω%).

Phase	Al	Mn	Fe	Ni	Cu
α	6.5–8.4	1	2.2–2.8	2.7–3.2	86
β	8.2–28.1	1.1–2.5	2–20	2.8–43.7	23.9–86
κ_Ⅰ_	9–14	1.36–3	46.9–72	3.5–16.2	9–21.6
κ_Ⅱ_	12–17.8	1.2–2.2	29.7–61	8–24.5	12.1–26.9
κ_III_	9–26.7	1–2	3–13.8	28.3–41.3	17–38.5
κ_Ⅳ_	10.5	2.4	73.4	7.3	6.6

**Fig. 4:**
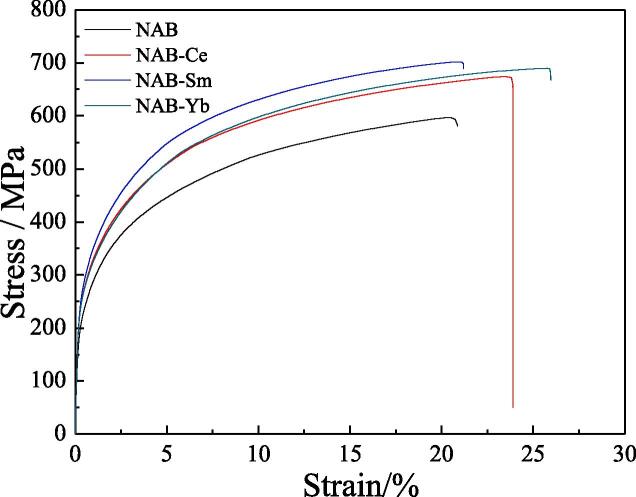
Mechanical properties of four NAB samples.

Fig. 3 shows that Ce or Sm or Yb has an evident effect on the metallographic structure of NAB alloy, and then they can directly affect the mechanical property of NAB alloy. As shown in Fig. 4, the tensile strength of NAB alloy without adding rare earth element is about 614 MPa, while the tensile strength of the NAB alloy with adding Ce or Sm or Yb reaches to 660 MPa, 708 MPa and 700 MPa, respectively. Meanwhile, the yield strength also increases and the elongation changes little with adding Ce or Sm or Yb. Clearly, the strength of NAB alloy increases with adding Ce or Sm or Yb, and the reason may be related to the decreased sizes of κ_Ⅰ_ and κ_Ⅱ_ phases. As showed in Fig. 3, κ phase dispersion distributes within the α phase and at the boundaries of the α phase with adding Ce or Sm or Yb, and the decreased size and the dispersion distribution of κ phase imply that the motion of the dislocation within α phase is prevented by the formation of dislocation pile up [32], [33], [34], [35], and then the mechanical property of NAB alloy increases with the addition of rare earth element.

### 3.2 Effects of Ce, Sm and Yb on the cavitation erosion of NAB alloy

**Fig. 5:**
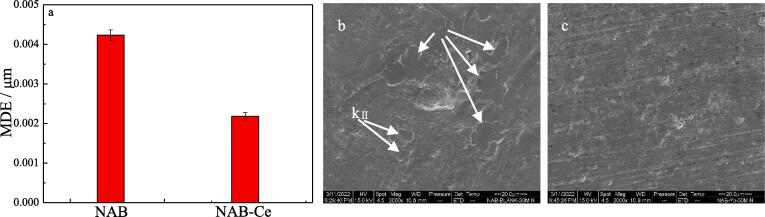
Mean depth of erosion (MDE, a) and the corresponding SEM images of NAB alloy (b) and NAB alloy containing Yb (c) after cavitation erosion for 30 min in 3.5% NaCl solution.

**Fig. 6:**
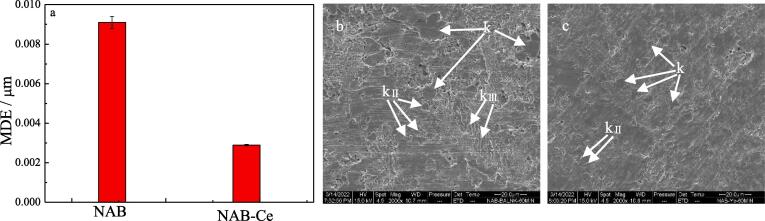
Mean depth of erosion (MDE, a) and the corresponding SEM images of NAB alloy (b) and NAB alloy containing Yb (c) after cavitation erosion for 60 min in 3.5% NaCl solution.

The result of the metallographic structure of NAB alloy demonstrates that adding Ce or Sm or Yb into NAB alloy can decrease the sizes of κ_Ⅰ_ and κ_Ⅱ_ phases, and then leading to the enhancement of the mechanical property of NAB alloy. Based on the previous papers [14], [15], [29], [30], [31], the boundaries between α and κ phase is the origin of the stress and crack caused by cavitation erosion, while the addition of the rare earth element can change the size of κ phase, and then it may affect the cavitation erosion of NAB alloy. Accordingly, Fig. 5, Fig. 6 show the mean depth of erosion (MDE) and the corresponding SEM images of NAB alloy and NAB alloy containing Yb alloy after cavitation erosion for 30 min and 60 min in 3.5% NaCl solution and in the case of without immersion, respectively.

Where MDE is usually calculated by dividing the measured mass loss by the density of the material to obtain the volume loss and dividing that by the area of the specified surface, and it can be expressed using the following equation [36]:

(1) $$MDE\left(\mu m\right)=\frac{\Delta W}{10\cdot \rho \cdot A}$$

where Δw refers to the mass loss caused by the cavitation erosion in mg, ρ is the sample density in g·cm^−3^, and A is the area of sample damaged by cavitation erosion in cm^2^.

It can be seen from Fig. 5a that MDE of NAB sample after cavitation erosion for 30 min is 0.00423 μm, and MDE decreases to 0.00218 μm when Yb is added into NAB alloy. Increasing cavitation erosion time to 60 min, and MDE of NAB sample increases to 0.0091 μm, while for MDE of NAB sample containing Yb, its MDE is 0.00289 μm. Evidently, MDE of NAB sample increases nearly twice with the increased cavitation erosion time, while MDE of NAB sample containing Yb changes slightly, and MDE of NAB sample containing Yb in the case of each cavitation erosion time is much lower than that of NAB sample, indicating the apparent improvement of the cavitation erosion resistance of NAB sample with the addition of Yb. Accordingly, Fig. 5b shows the SEM image of NAB sample after cavitation erosion for 30 min, it clearly shows that the initial cracks occur at the boundaries between α and κ phases (κ_Ⅰ_ and κ_Ⅱ_ phases). As the addition of Yb significantly decreases the sizes of κ_Ⅰ_ and κ_Ⅱ_ phases, the boundary volume of the grain boundary between α phase and κ phase decreases, and then the crack growth caused by cavitation erosion is greatly prevented at the grain boundary between α phase and κ phase, and finally resulting in the decreased MDE at the initial stage of cavitation erosion.

Increasing cavitation erosion time to 60 min, as shown in Fig. 6b, a significant mass loss occurs at the grain boundary between α phase and κ phase (κ_Ⅰ_ and κ_Ⅱ_ phases), and it is further verified that the boundary between α phase and κ phase is the source of cracks caused by cavitation erosion. While for NAB sample containing Yb, the surface roughness significantly decreases (see Fig. 6c), meaning the reduced cavitation erosion damage. Moreover, it is clearly seen from Fig. 6c that the sizes of κ_Ⅰ_ and κ_Ⅱ_ phases evidently decrease, and then the propagation of cracks caused by cavitation erosion is effectively inhibited at the grain boundary between α phase and κ phase, and finally the cavitation erosion resistance of NAB alloy containing Yb is higher than that of NAB alloy.

**Fig. 7:**
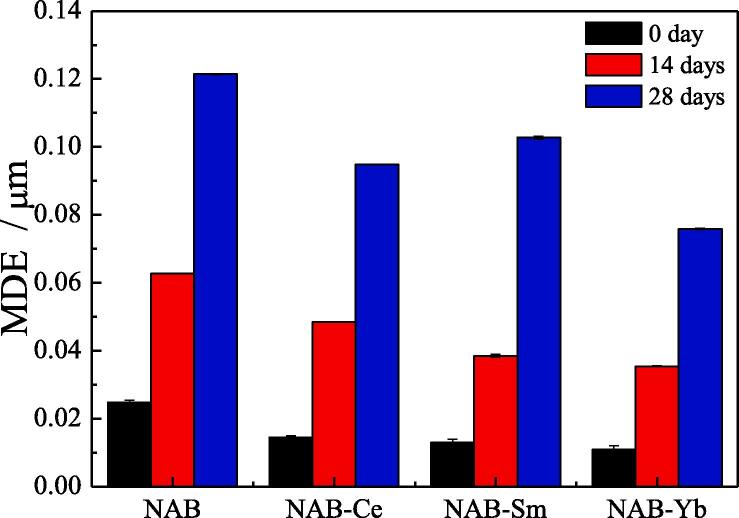
MDE of four samples after cavitation erosion for 2 h in 3.5% NaCl solution and in the case of different immersion time.

Before discussing the effect of immersion time on the cavitation erosion of NAB alloy whether containing rare earth element or not, it is necessary to point out that four NAB samples are firstly immersed in 3.5% NaCl solution for 14 days and 28 days, respectively. After immersion, the cavitation erosion experiment is immediately carried out, and the cavitation erosion experiment of four NAB samples without immersion (0 day) is also carried out for comparison. The influence of Ce or Sm or Yb on MDE of NAB sample is showed in Fig. 7, it can be seen that MDE after cavitation erosion for 2 h in 3.5% NaCl solution increases with increasing the immersion time in the case of each NAB sample, which implies that the increased immersion time can aggravate the cavitation erosion damage of NAB alloy in 3.5% NaCl solution, and the reason may be related to the synergy of cavitation erosion and corrosion. While for NAB alloy without adding rare earth element, MDE reaches to 0.02478 μm in the case of without immersion (0 day), which is 1.713 times, 1.913 and 2.253 times higher than that of NAB sample containing Ce or Sm or Yb in the case of without immersion (0 day), respectively. Increasing immersion time to 14 days, MDE of NAB sample increases from 0.02478 μm to 0.06273 μm, which is 1.295, 1.629 and 1.77 times higher than MDE of NAB alloy containing Ce or Sm or Yb, respectively. Similarly, MDE of four samples continuously increases with increasing immersion time to 28 days, and MDE of NAB alloy containing Ce or Sm or Yb is 76.23%, 84.6% and 62.5% of MDE of NAB alloy. Evidently, adding Ce or Sm or Yb into NAB alloy can significantly decrease MDE of NAB alloy in 3.5% NaCl solution, that is adding Ce or Sm or Yb into NAB alloy can increase the cavitation erosion resistance of NAB alloy in 3.5% NaCl solution.

The above MDE results reveal that the cavitation erosion resistance of NAB alloy evidently increases with adding Ce or Sm or Yb, and Fig. 3 shows that the size of κ phase decreases significantly with the addition of Ce or Sm or Yb, which means that the fine grain size of κ phase is more resistant to cavitation erosion. The dependence of cavitation erosion resistance on the size of κ phase of NAB alloy can be explained by the decreased stress, crack and corrosion at the boundaries between α and κ phases. As noted above, the stress caused by cavitation erosion and the corrosion caused by the potential difference between α and κ phases are reduced with the decreased size of κ phase, and then, the initiation and propagation of cracks caused by cavitation erosion are significantly slowed down with the decreased size of κ phase, and the corrosion trend at the boundary between α and κ phases is correspondingly slowed down, and finally leading to the increased cavitation erosion and corrosion resistances of NAB alloy.

**Fig. 8:**
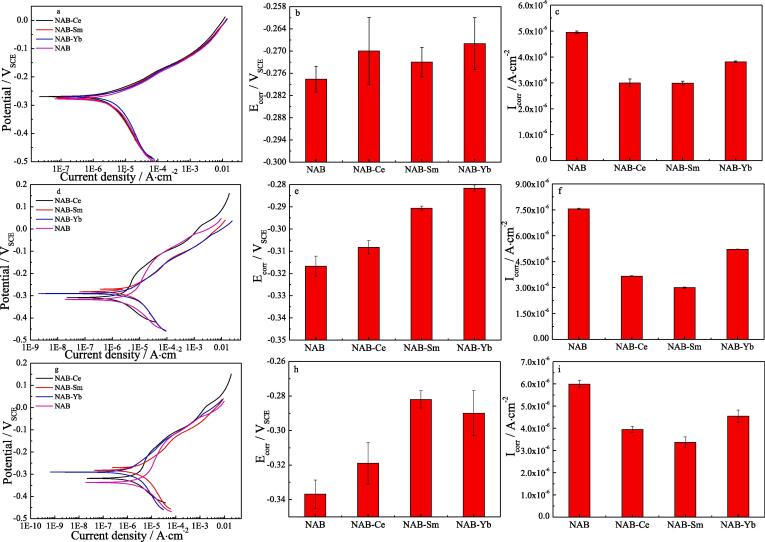
Tafel plots and the corresponding corrosion parameters of four NAB samples in 3.5% NaCl solution and in the case of different immersion time, a), b) and c) 0 day, d), e) and f) 14 days, g), h) and i) 28 days.

Besides the reason of the reduced stress and crack at the grain boundaries between α and κ phases, another reason of the increased cavitation erosion resistance of NAB alloy with adding rare earth element may be related to the increased corrosion resistance of NAB alloy with adding rare earth element. Similar to the cavitation erosion experiment, four NAB samples are firstly immersed in 3.5% NaCl solution for 0 day (without immersion), 14 days and 28 days, respectively. After immersion, the electrochemical experiments are immediately carried out in 3.5% NaCl solution. Tafel plots of four NAB samples after immersion for 0 day, 14 days and 28 days in 3.5%% NaCl solution are displayed in Fig. 8, it can be found that the corrosion potential of NAB alloy in 3.5% NaCl solution moves to positive direction and the corrosion current density decreases with the addition of Ce or Sm or Yb in the case of each immersion time, indicating the enhanced corrosion resistance of NAB alloy. The corrosion parameters extracted from Fig. 8a, 8c and 8e are presented in Fig. 8b, 8d and 8f, and it reveals that E_corr_ moves to negative direction and i_corr_ increases with prolonging the immersion time, indicating the decreased corrosion resistance of NAB alloy. Moreover, E_corr_ moves to positive direction and i_corr_ decreases with adding rare earth element in the case of each immersion time, meaning the improvement of corrosion resistance of NAB alloy with adding rare earth element. The increment of MDE and the corrosion resistance with the prolonged immersion time indicates that the increased corrosion may promote the cavitation erosion to a large extent, and this result is consistent with the synergistic effect of cavitation erosion and corrosion reported in literature [37], [38], [39], [40], [41], [42].

### 3.3 Surface morphology of specimen after cavitation erosion

**Fig. 9:**
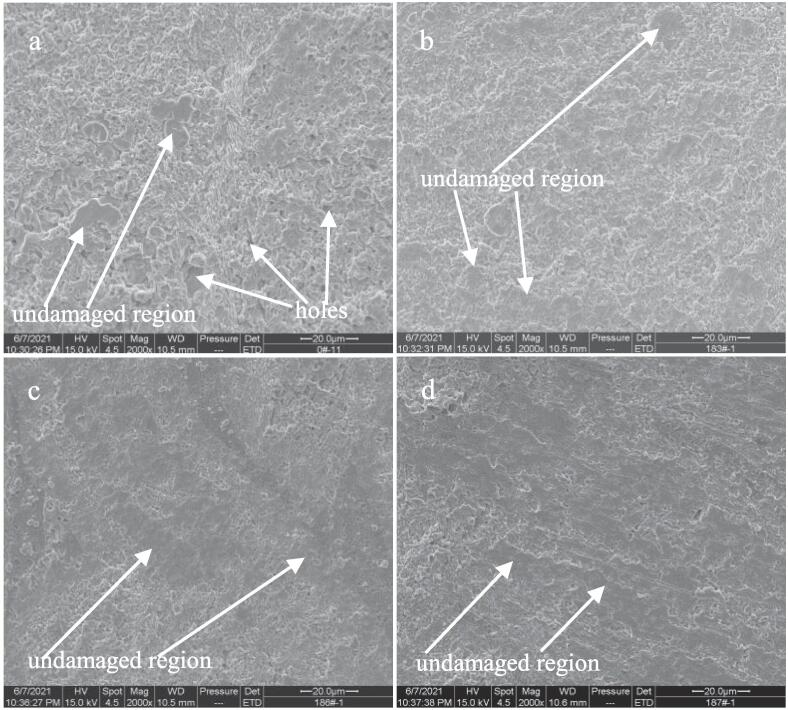
Front SEM images of NAB alloys after cavitation erosion for 2 h in 3.5% NaCl solution and in the case of without immersion (0 day), a) NAB alloy, b) NAB-Ce alloy, c) NAB-Sm alloy, d) NAB-Yb alloy.

In order to insight into the effects of Ce, Sm and Yb on the cavitation erosion behavior of NAB alloy in 3.5% NaCl solution, the SEM images of four samples after cavitation erosion for 2 h are observed. As shown in Fig. 9a, the surface of NAB sample becomes very coarse after cavitation erosion for 2 h in the case of without immersion (0 day), and some holes can be observed, indicating an evident mass loss. Even so, a little of undamaged regions by cavitation erosion on the surface can still be found. While for NAB sample containing Ce (see Fig. 9b), the number and size of holes on the surface are significantly reduced, and the area of the un-damaged region by cavitation erosion clearly increases, which means that the cavitation erosion damage of NAB sample containing Ce is much lighter than that of NAB sample. Similarly, the areas of the un-damaged regions on the surfaces of NAB sample containing Sm and NAB sample containing Yb are further increased, and the surface roughness also decreases significantly, demonstrating that the cavitation erosion damage degree of NAB sample containing Sm or Yb is significantly lighter than that of NAB sample.

**Fig. 10:**
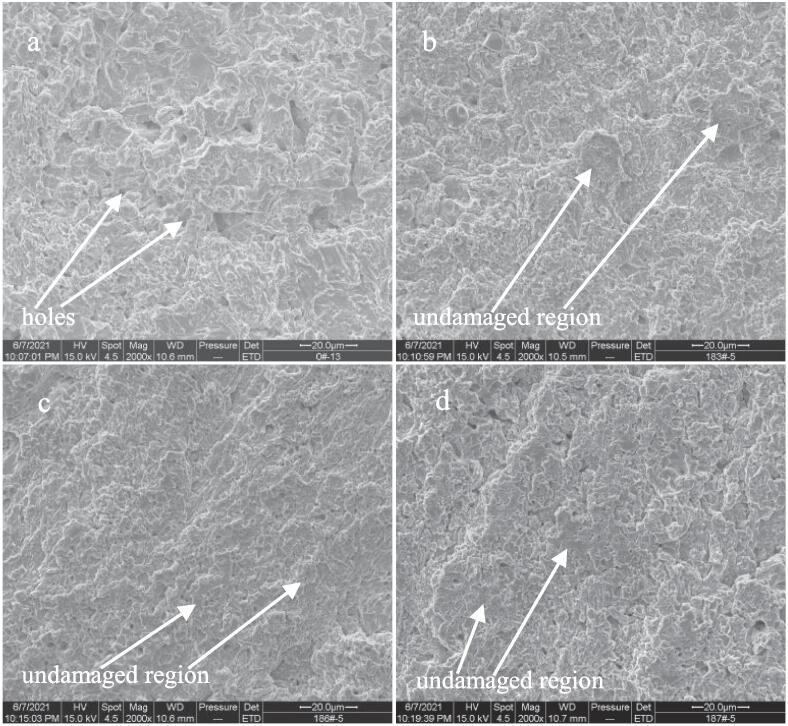
Front SEM images of NAB alloys after cavitation erosion for 2 h in 3.5% NaCl solution and in the case of immersion for 14 days, a) NAB alloy, b) NAB-Ce alloy, c) NAB-Sm alloy, d) NAB-Yb alloy.

Increasing the immersion time to 14 days, the damage caused by cavitation erosion on each NAB sample surface is evidently aggravated by comparing with each NAB sample surface without immersion, and the reason may be related to the promotion of the cavitation erosion by corrosion. As shown in Fig. 10a, the size of the hole on the surface of NAB sample significantly enlarges, and the number of the hole increases by comparing with Fig. 9a. Meanwhile, the surface roughness sharply increases, and the un-damaged regions thoroughly disappear, indicating the aggravated cavitation erosion damage with increasing immersion time to 14 days. While for NAB sample containing Ce (see Fig. 10b), Sm (see Fig. 10c) and Yb (see Fig. 10d), the surface damage relatively reduces by comparing with NAB sample surface, i.e., the surface roughness evidently decreases and some un-damaged areas can still be found on sample surface, implying the significant improvement of the cavitation erosion resistance of NAB alloy with the addition of Ce or Sm or Yb in 3.5% NaCl solution.

**Fig. 11:**
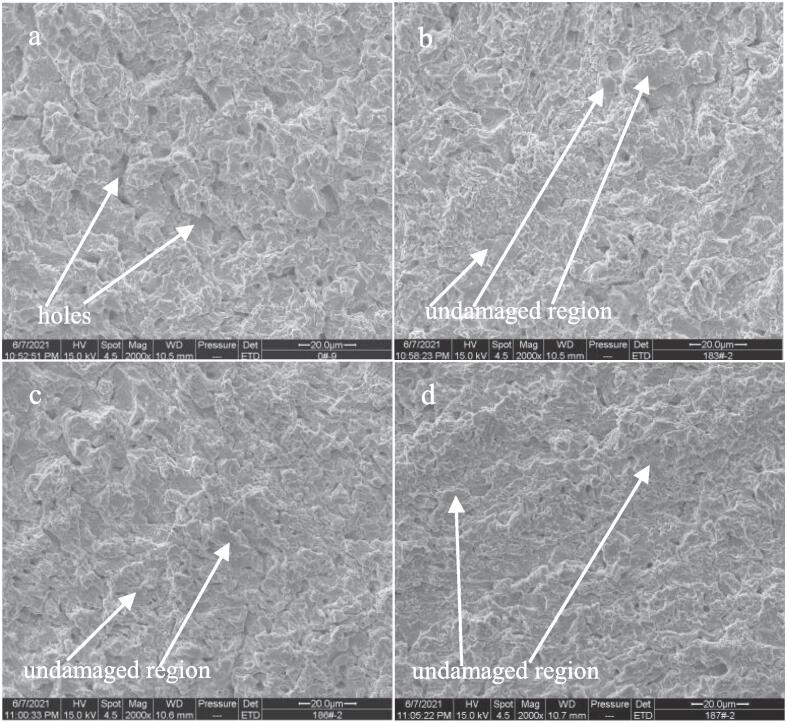
Front SEM images of NAB alloys after cavitation erosion for 2 h in 3.5% NaCl solution and in the case of immersion for 28 days, a) NAB alloy, b) NAB-Ce alloy, c) NAB-Sm alloy, d) NAB-Yb alloy.

Continuously increasing the immersion time to 28 days, the surface damage caused by cavitation erosion on each NAB sample surface is further aggravated by comparing with each NAB sample surface in the case of immersion for 14 days. As shown in Fig. 11a, large holes and evident mass loss can be easily observed on the surface of NAB sample, implying the serious damage caused by cavitation erosion. Fig. 11b, 11c and 11d show that the large holes and significant mass loss can still be found on the surface of NAB sample containing Ce or Sm or Yb, however, the surface roughness of each NAB sample containing rare earth element becomes smaller than that of NAB sample, and even a small amount of undamaged regions can be visible, demonstrating the evident alleviation of the damage caused by cavitation erosion. On the whole, it can be concluded from the above SEM images that the damage of NAB sample surface reduces with the addition of Ce or Sm or Yb.

**Fig. 12:**
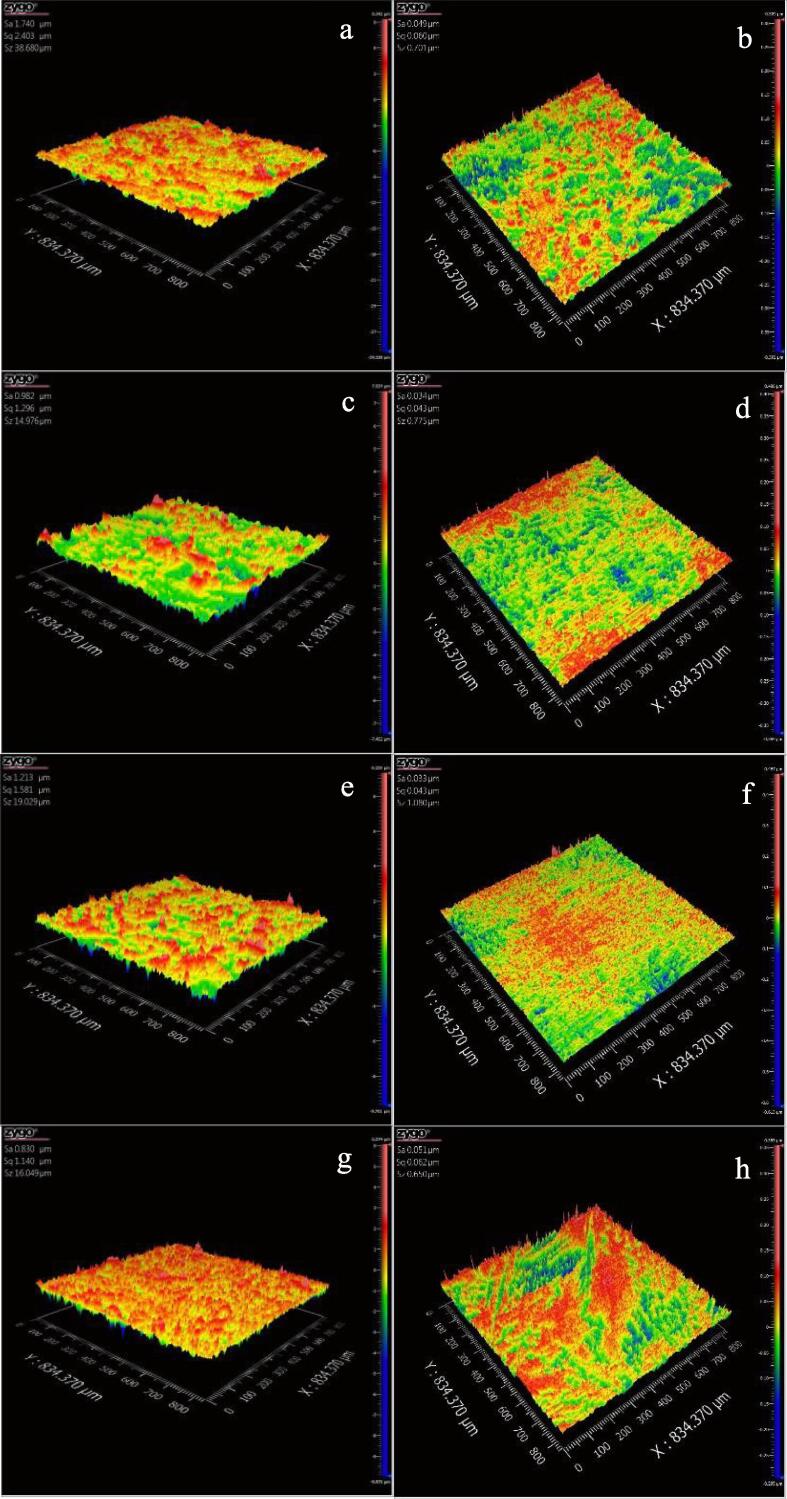
3D morphologies of NAB alloys before and after cavitation erosion for 2 h in 3.5% NaCl solution and in the case of without immersion (0 day), a) NAB alloy after cavitation erosion, b) NAB alloy before cavitation erosion, c) NAB-Ce alloy after cavitation erosion, d) NAB-Ce alloy before cavitation erosion, e) NAB-Sm alloy after cavitation erosion, f) NAB-Sm alloy before cavitation erosion, g) NAB-Yb alloy after cavitation erosion, h) NAB-Yb alloy before cavitation erosion.

**Fig. 13:**
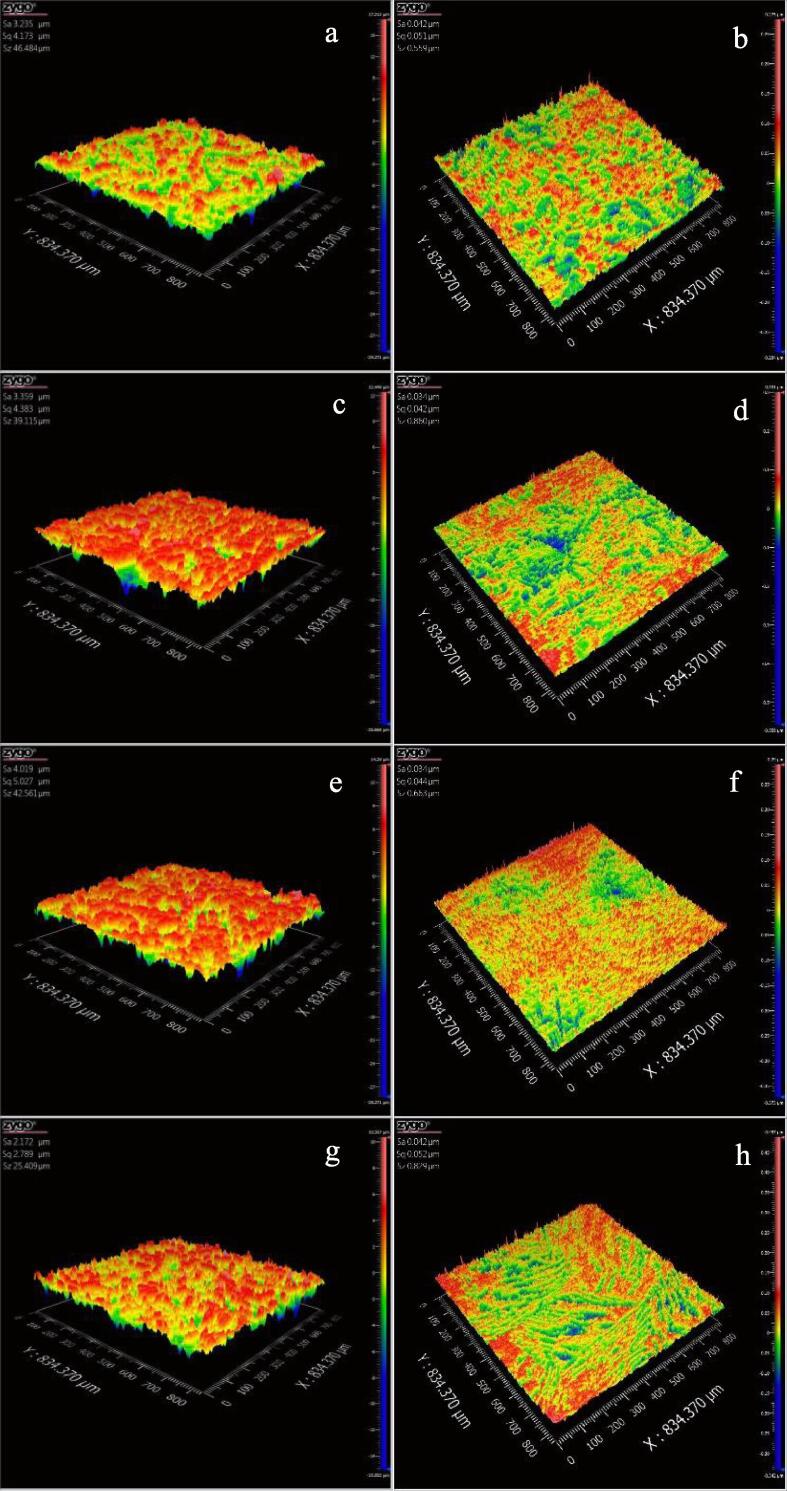
3D morphologies of NAB alloys before and after cavitation erosion for 2 h in 3.5% NaCl solution and in the case of immersion for 14 days, a) NAB alloy after cavitation erosion, b) NAB alloy before immersion, c) NAB-Ce alloy after cavitation erosion, d) NAB-Ce alloy before immersion, e) NAB-Sm alloy after cavitation erosion, f) NAB-Sm alloy before immersion, g) NAB-Yb alloy after cavitation erosion, h) NAB-Yb alloy before immersion.

**Fig. 14:**
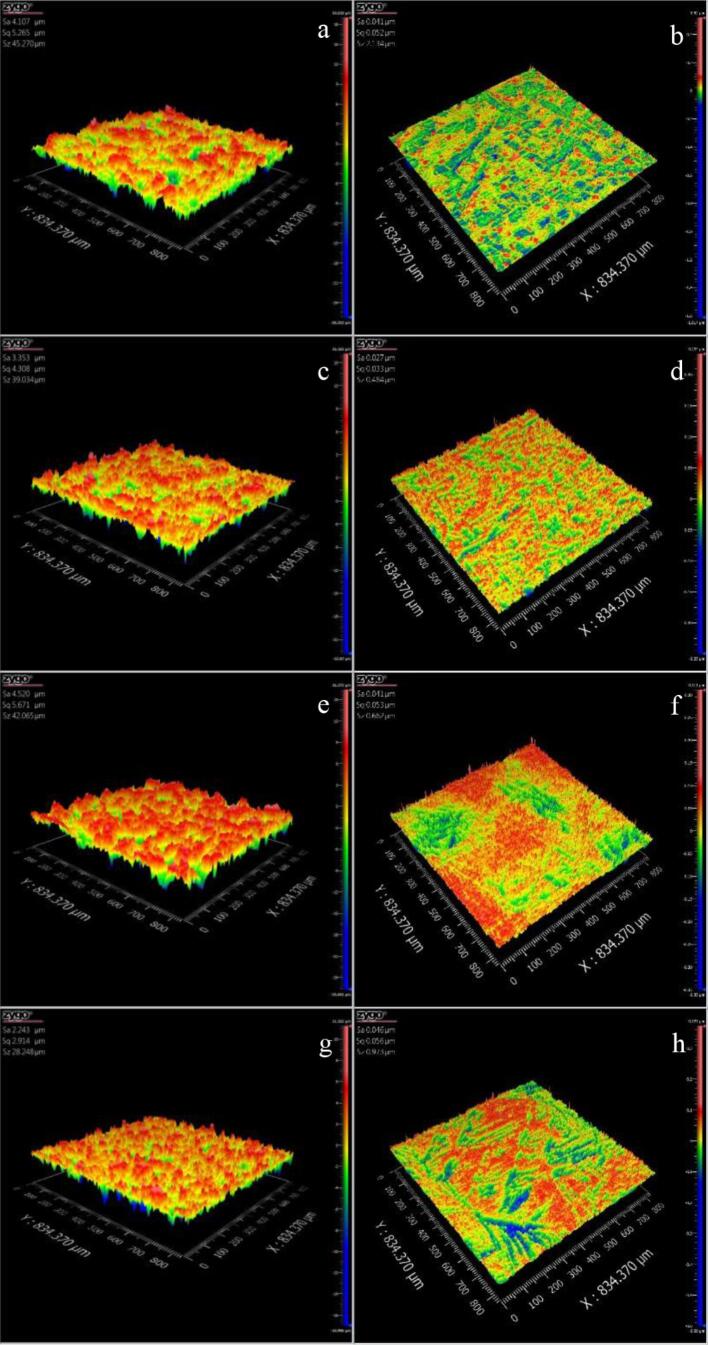
3D morphologies of NAB alloys before and after cavitation erosion for 2 h in 3.5% NaCl solution and in the case of immersion for 28 days, a) NAB alloy after cavitation erosion, b) NAB alloy before immersion, c) NAB-Ce alloy after cavitation erosion, d) NAB-Ce alloy before immersion, e) NAB-Sm alloy after cavitation erosion, f) NAB-Sm alloy before immersion, g) NAB-Yb alloy after cavitation erosion, h) NAB-Yb alloy before immersion.

In order to further analyze the effects of Ce, Sm and Yb on the cavitation corrosion behavior of NAB alloy in 3.5% NaCl solution, Fig. 12 shows the 3D images of the damaged morphology of four NAB samples before and after cavitation erosion for 2 h in 3.5% NaCl solution in the case of without immersion (0 day), respectively. Clearly, Fig. 12b, 12d, 12f and 12 h show that the surface roughness and Z-axis of each NAB sample before cavitation erosion are almost identical. Similarly, Fig. 13b, 13d, 13f, 13 h, 14b, 14d, 14f and 14 h display that the surface states of four NAB samples before immersion are almost consistent. Fig. 12a shows that the surface of NAB sample is uneven comparing with the evident mass loss, and some small holes are also visible, indicating the significant damage caused by cavitation erosion. While Fig. 12c, 12e and 12 g show that the surfaces of three NAB samples containing Ce, Sm and Yb become smoother than that of NAB sample, and the changes of Z-axis and surface roughness are smaller, indicating the alleviation of the damage caused by cavitation erosion.

Increasing the immersion time to 14 days, Fig. 13a shows that the surface of NAB sample after cavitation erosion for 2 h becomes rougher, and the surface roughness further increases by comparing with Fig. 12a, implying the aggravated damage caused by cavitation erosion. Similarly, the surface of NAB sample containing Ce or Sm or Yb becomes smoother than that of NAB sample (see Fig. 13c, 13e and 13g), but its surface roughness is higher than that showed in Fig. 12b, 12c and 12d, indicating the alleviation of the damage caused by cavitation erosion with the addition of the rare earth element and the aggravated damage with the prolonged immersion time.

Continuously increasing immersion time to 28 days, as shown in Fig. 14a, the Z-axis and the surface roughness of each NAB sample surface continuously increase, big holes and the serious uneven regions are more visible, which implies that the damage caused by cavitation erosion further aggravates with immersion time. While Fig. 14c, 14e and 14g reveal that whether the size and deep of the hole or the Z-axis and the surface roughness of NAB sample containing Ce or Sm or Yb decrease by comparing with Fig. 14a, demonstrating the alleviation of the damage caused by cavitation erosion with adding Ce or Sm or Yb.

## 4 Conclusions

NAB alloy and NAB alloy containing Ce or Sm or Yb are manufactured in this paper, and their cavitation erosion behaviors in 3.5% NaCl solution are investigated. Based on the experimental results, some conclusions can be drawn as following:

1) The origin of cavitation erosion originates from the boundaries between α phase and κ phase, the small size of κ phase can decrease the boundary volume between α phase and κ phase, and then the growth and propagation of crack caused by cavitation erosion at the grain boundary between α phase and κ phase are inhibited.
2) The addition of Ce or Sm or Yb into NAB alloy can significantly decrease the sizes of κ_Ⅰ_ and κ_Ⅱ_ phases, and thereby increasing the resistance of crack growth caused by cavitation erosion at the grain boundary between α phase and κ phase, and finally resulting in the increased cavitation erosion resistance.
3) The corrosion resistance of NAB alloy containing rare earth element or not in 3.5% NaCl solution decreases with the increased immersion time, and the decrement of the corrosion resistance may promote the damage caused by cavitation erosion. Ce or Sm or Yb can increase the corrosion resistance of NAB alloy in 3.5% NaCl solution, and then resulting in the increased cavitation erosion resistance of NAB alloy.

## Declaration of Competing Interest

The authors declare that they have no known competing financial interests or personal relationships that could have appeared to influence the work reported in this paper.
